# Mouse cortical organoids reveal key functions of p73 isoforms: TAp73 governs the establishment of the archetypical ventricular-like zones while DNp73 is central in the regulation of neural cell fate

**DOI:** 10.3389/fcell.2024.1464932

**Published:** 2024-09-23

**Authors:** Hugo Alonso-Olivares, Margarita M. Marques, Anna Prieto-Colomina, Lorena López-Ferreras, Nicole Martínez-García, Alberto Vázquez-Jiménez, Victor Borrell, Maria C. Marin, Rosalia Fernandez-Alonso

**Affiliations:** ^1^ Instituto de Biomedicina and Departamento de Biología Molecular, Universidad de León, León, Spain; ^2^ Instituto de Desarrollo Ganadero y Sanidad Animal and Departamento de Producción Animal, Universidad de León, León, Spain; ^3^ Instituto de Neurociencias, Consejo Superior de Investigaciones Científicas and Universidad Miguel Hernández, Alicante, Spain; ^4^ Instituto de Biomedicina and Departamento de Producción Animal, Universidad de León, León, Spain

**Keywords:** mouse brain organoids, brain development, brain morphogenesis, p53-family, p73, TAp73, DNp73, neurogenesis

## Abstract

**Introduction:**

Neurogenesis is tightly regulated in space and time, ensuring the correct development and organization of the central nervous system. Critical regulators of brain development and morphogenesis in mice include two members of the p53 family: p53 and p73. However, dissecting the *in vivo* functions of these factors and their various isoforms in brain development is challenging due to their pleiotropic effects. Understanding their role, particularly in neurogenesis and brain morphogenesis, requires innovative experimental approaches.

**Methods:**

To address these challenges, we developed an efficient and highly reproducible protocol to generate mouse brain organoids from pluripotent stem cells. These organoids contain neural progenitors and neurons that self-organize into rosette-like structures resembling the ventricular zone of the embryonic forebrain. Using this model, we generated organoids from p73-deficient mouse cells to investigate the roles of p73 and its isoforms (TA and DNp73) during brain development.

**Results and Discussion:**

Organoids derived from p73-deficient cells exhibited increased neuronal apoptosis and reduced neural progenitor proliferation, linked to compensatory activation of p53. This closely mirrors previous *in vivo* observations, confirming that p73 plays a pivotal role in brain development. Further dissection of p73 isoforms function revealed a dual role of p73 in regulating brain morphogenesis, whereby TAp73 controls transcriptional programs essential for the establishment of the neurogenic niche structure, while DNp73 is responsible for the precise and timely regulation of neural cell fate. These findings highlight the distinct roles of p73 isoforms in maintaining the balance of neural progenitor cell biology, providing a new understanding of how p73 regulates brain morphogenesis.

## 1 Introduction

The mammalian brain is an extremely complex structure that relies heavily on the cerebral cortex for higher cognitive function (Borrell, 2019). The development of the cerebral cortex begins with the amplification of neuroepithelial stem cells, which then transition into apical Radial Glial Cells (aRGCs), initiating neurogenesis. aRGCs are the primary type of cortical progenitor cells, constituting the primary germinal layer: the Ventricular Zone (VZ). In rodents, aRGCs primarily undergo symmetric proliferative divisions at the onset of neurogenesis, leading to the expansion of their pool. As development proceeds, aRGC self-amplification decreases with the concomitant increase in asymmetric divisions, giving rise to secondary, basal progenitor cells (indirect neurogenesis) and neurons (direct neurogenesis) (Miyata et al., 2004; Takahashi et al., 1995; Takahashi et al., 1996). Basal progenitors migrate from the apical surface to the basal border of the VZ, forming the Subventricular Zone (SVZ), a secondary germinal zone. In rodents, the SVZ is a significant neurogenic niche in the adult brain, where predominantly excitatory neurons for all cortical layers are generated (Miyata et al., 2004; Noctor et al., 2004; Noctor et al., 2008; Taverna et al., 2014). Towards the end of neurogenesis, most aRGCs differentiate into glial or ependymal cells, depleting the neural stem cell (NSC) pool and marking the end of neurogenesis (Villalba et al., 2021). The neurogenic capacity and homeostasis of the rodents SVZ is dependent on the integrity of the ependymal cells and their capacity to assemble into the unique structures that define this region: the pinwheels (Kuo et al., 2006; Paez-Gonzalez et al., 2011).

The p53 family of transcription factors is involved in several developmental processes (Maeso-Alonso et al., 2021; Van Nostrand et al., 2017), including the regulation of morphogenesis and function of the central nervous system (CNS) (Agostini et al., 2010; Fujitani et al., 2010; Gonzalez-Cano et al., 2010; Hooper et al., 2006; Mendrysa et al., 2011; Moreau et al., 2023; Talos et al., 2010; Xiong et al., 2020). Among the p53 family members, p73 is considered a key regulator of CNS development and function. Indeed, p73 knockout (p73KO) mice exhibit a plethora of severe developmental defects (Marques et al., 2019; Nemajerova and Moll, 2019; Rufini et al., 2011) including anomalies in the organization and homeostasis of the neurogenic niche of the SVZ (Gonzalez-Cano et al., 2016). Global p73KO mice display hippocampal dysgenesis characterized by a missing or truncated dentate gyrus, cortical thinning, loss of sympathetic and Cajal-Retzius neurons and enlarged ventricles with hydrocephalus (Medina-Bolivar et al., 2014; Meyer et al., 2004; Yang et al., 2000). Moreover, p73 deficiency results in deficient ependyma that fails to assemble into neurogenic pinwheels and consequently to organize a functional SVZ niche (Gonzalez-Cano et al., 2016). In mice, p73 is necessary to maintain self-renewal and proliferation, and to inhibit senescence of NSCs or neural progenitor cells (NPCs), therefore maintaining the neurogenic pool (Agostini et al., 2010; Fujitani et al., 2010; Gonzalez-Cano et al., 2010; Talos et al., 2010). However, despite strong *in vivo* and *in vitro* evidence supporting the relevance of p73 in neural development, the specific cellular and molecular mechanisms of action remain unknown.

The *Trp73* gene gives rise to two different isoforms, TAp73 and DNp73, which are typically expressed in a tissue specific manner (Grespi et al., 2012). TAp73 is transcriptionally competent, while the N-terminal truncated DNp73 acts as dominant-negative of both p53 and TAp73; however, DNp73 has its own unique functions (Marques-Garcia et al., 2009; Niemantsverdriet et al., 2012). Consequently, the activity and function of p73 result from a context-dependent balance between its isoforms. The brain phenotypes observed in p73 isoform-specific knockout mice are milder compared to those in global p73KO mice. DNp73 knockout (DNp73KO) mice exhibit neuronal loss (Tissir et al., 2009; Wilhelm et al., 2010), while TAp73 knockout (TAp73KO) mice display hippocampal dysgenesis (Fujitani et al., 2010; Tomasini et al., 2008). Notably, some phenotypic features present in p73KO mice do not manifest in any of the isoform-specific knockouts. These findings suggest that complex interactions between the p73 isoforms play a crucial role in brain development and morphogenesis, which are yet to be fully understood.

It is well established that inherent differences exist between the *in vivo* models and the traditional 2D *in vitro* systems (Gopalakrishnan, 2019), highlighting the need for multi-system strategies to generate comprehensive datasets and dissect the molecular and cellular function of p73 in neural development and brain morphogenesis. Brain organoids, defined as complex 3D structures that develop from stem cells through a self-organization process, have emerged as powerful *in vitro* tools for studying brain cellular morphogenesis, structure and microenvironment (Pasca et al., 2022; Xu et al., 2021). In this work, we present an improved, robust and cost-effective model of mouse brain organoids (mBOs). Leveraging this protocol, we have generated mBOs to study the role of p53 and p73 isoforms in mouse neurodevelopment.

Our results reveal specific functions of p73 isoforms, with TAp73 governing transcriptional programs related to cell adhesion, extracellular matrix organization and epithelial cell morphogenesis, which are essential for establishing the archetypical proliferative ventricular-like zones in mBOs, resembling the mouse neurogenic niches. On the other hand, DNp73 regulates the early neuroectodermal fate switch and is thus crucial for the precise regulation of neural cell fate, as well as for preserving the undifferentiated and proliferative state of the NSC pool.

## 2 Materials and methods

### 2.1 Cell culture

Four mouse induced pluripotent stem cell (miPSC) lines were used (2 clones per line): (i) wild-type (WT), (ii) p73KO, (iii) p53 knockout (p53KO) and (iv) p73KO/p53KO (DKO)-miPSCs, whose generation and culture have been described previously (Martin-Lopez et al., 2017). Briefly, miPSCs were cultured on mouse embryonic fibroblast feeder cells (5 × 10^4^ cells/cm^2^) in Dulbecco’s Modified Eagle Medium (DMEM, Cat# D5671, Sigma-Aldrich, MO, United States) supplemented with 15% fetal bovine serum (FBS, Cat# 17479633, Thermo Fisher Scientific, MA, United States), 2 mM L-glutamine (Cat# 7513, Sigma-Aldrich, MO, United States), 1 mM sodium pyruvate (Cat# 11360039, Thermo Fisher Scientific, MA, United States), 1 mM nonessential amino acids (Cat# 11140035, Thermo Fisher Scientific, MA, United States), 0.1 mM β-mercaptoethanol (Cat# M3148, Sigma-Aldrich, MO, United States) and 1,000 U/mL Leukemia Inhibitory Factor (LIF, Cat# ESG1107, Millipore, MA, United States).

Also, the following mouse embryonic stem cell (mESC) lines were used: (i) parental E14TG2α (Hooper et al., 1987) were kindly provided by Dr. Jim McWhir (former Researcher at the Roslin Institute, Edinburgh, Scotland, United Kingdom); (ii) E14-TAp73KO (3 clones) and (iii) E14-DNp73KO cells (3 clones), which were generated in our lab (Lopez-Ferreras et al., 2021). These cells were cultured on 0.1% gelatin-coated plates (10^5^ cells/cm^2^) in Glasgow Minimum Essential Medium (GMEM, Cat# G-5154, Sigma-Aldrich, MO, United States) supplemented with 10% FBS, 2 mM L-glutamine, 1 mM sodium pyruvate, 1 mM nonessential amino acids, 0.1 mM β-mercaptoethanol and 500 U/mL LIF.

### 2.2 Mouse brain organoid generation

For mBOs generation, mPSCs were dissociated to single cells using 0.25% trypsin-EGTA and 8 × 10^3^ cells were seeded and quickly aggregated in each well of 96-well ultra-low attachment plates (Cat# 7007, Costar, ME, United States), in differentiation medium (100 μL/well) containing GMEM supplemented with 10% KnockOut Serum Replacement (KSR, Cat# 10828010, Gibco, MA, United States), 2 mM GlutaMAX (Cat# 35050038, Gibco, MA, United States), 1 mM sodium pyruvate, 0.1 mM nonessential amino acids and 0.1 mM β-mercaptoethanol. A partial medium change (half of the volume) was performed on day 4, and 3 days later, cell aggregates were transferred to a 10-cm bacterial-grade dish in N2 medium: DMEM/F12 (Cat# D8437, Sigma-Aldrich, MO, United States) supplemented with 1:100 N2 (Cat# 17502–048, Gibco, MA, United States) and GlutaMAX. The medium was changed every 3 days until day 14. The day on which mPSCs were seeded in ultra-low attachment plates to differentiate is referred to as differentiation day 0.

The initial optimization of the protocol was performed using the E14TG2⍺ mESC line. We conducted several rounds of experiments, introducing stepwise modifications. These changes were systematically compared to the efficiency of the reference protocol developed by Eiraku et al. (2008), allowing us to iteratively refine and enhance our method. After optimization, we successfully generated mBOs across seven independent batches of mESCs. To further validate the robustness of our protocol, we extended its application to miPSCs, where we conducted experiments with four additional batches.

### 2.3 Tissue processing

mBOs were fixed in 4% paraformaldehyde (PFA) containing 6% sucrose at 4°C for 30 min. After fixation, mBOs were washed with PBS, cryoprotected overnight with 30% sucrose and stored at 4°C in PBS. Then, mBOs were embedded in PolyFreeze Tissue Freezing Medium (Cat# SHH0026, Sigma-Aldrich, MO, United States) and frozen in Peel-A-Way^™^ embedding molds (Cat# 18986-1, Polysciences, PA, United States) using dry ice. mBOs were finally cryosectioned at 8 μm using a Leica cryostat (Cat# CM 1950, Leica, Germany) and the obtained sections were collected in SuperFrost Plus™ Adhesion slides (Cat# J1800AMNZ, Epredia, NH, United States) and stored at −80°C.

### 2.4 Immunofluorescence

mBO cryosections were permeabilized in PBS containing 0.25% Triton X-100 and blocked in 10% of horse serum and 2% bovine serum albumin (BSA) for 2 h at room temperature (RT). mBO slices were incubated with primary antibodies overnight at 4°C in blocking solution, followed by the appropriate fluorophore-conjugated secondary antibodies incubation for 2 h. Nuclei were counterstained with DAPI.

Primary antibodies used were: anti-SOX2 (1:200, goat polyclonal, Cat# AF 2018, R&D Systems, MN, United States); anti-bIII tubulin (1:1,000, mouse monoclonal, Cat# MMS-435P, Covance, NJ, United States); anti-N-Cadherin (1:200, rabbit polyclonal, Cat# 13116, Cell Signaling, MA, United States); anti-NeuN (1:200, mouse monoclonal, Cat# MAB377, Merck Millipore, MA, United States); anti-phospho Histone H3 (pHH3) (1:500, mouse monoclonal, Cat# 9706, Cell Signaling); anti-Cleaved Caspase-3 (CC3) (1:200, rabbit polyclonal, Cat# 9664, Cell Signaling). The secondary antibodies were: Alexa568 anti-goat (Cat# A11057, Invitrogen, MA, United States); Alexa647 anti-mouse (Cat# A31571, Invitrogen); Alexa488 anti-rabbit (Cat# A21206, Invitrogen); Alexa647 anti-mouse IgG2a (Cat# A21241, Invitrogen); Alexa594 anti-mouse IgG1 (Cat# A21125, Invitrogen); all diluted 1:1,000.

### 2.5 Image acquisition, analysis and quantification

Confocal microscopy images (8 bits) were obtained in a Zeiss LSM800 Confocal Laser Scanning Microscope (Carl Zeiss Microscopy GmbH, Germany) using 25 × Plan-Apo/0.8 numerical aperture or 63 × Plan-Apo/1.4 numerical aperture oil objectives at RT. In some cases, a 0.5 × digital zoom was used. Confocal Z-stack images were acquired, and stacks were z-projected to maximum intensity. Images were processed with the ZEN blue software (Carl Zeiss Microscopy GmbH). All images were further analyzed using ImageJ/Fiji software (MD, United States). Three batches of mESCs-derived organoids and another three batches generated from miPSCs were used for quantifications. For each genotype, five different mBO cryosections *per* clone were analyzed across the three independent batches resulting in a total number of, at least, 15 mBOs *per* condition.

For the quantification of the number of ventricles, 25 × 0.5 Z-stack confocal images of mBO slices were employed to manually count the number of ventricles in each mBO cryosection based on the presence of SOX2+ neural rosettes with a defined N-CAD+ lumen.

For the quantification of NPCs, 63 × Z-stack confocal images were used and the number of SOX2+ and the total number of cells (DAPI-stained nuclei) were manually counted. Also, NeuN+ cells were manually quantified to calculate the percentage of NeuN+ cells.

The percentage of proliferating or apoptotic cells was calculated by analyzing 63 × Z-stack pictures to manually count the number of pHH3+ or CC3+ cells and the total number of cells (DAPI-stained nuclei). To quantify the percentage of proliferating progenitors and apoptotic progenitors or neurons, colocalization studies were performed using 63 × Z-stack confocal images. Images of the pHH3 or the CC3 channel were binarized using an automatic threshold. A selection was created (ROI) and applied to the SOX2 or TUJ1 channel. Then, the “Clear Outside” plug-in was applied to eliminate the signal without double staining. Finally, cells co-expressing pHH3/SOX2, CC3/SOX2 or CC3/TUJ1 were counted using ImageJ/Fiji.

### 2.6 RNA sequencing and transcriptome data analysis

Bulk RNA from WT-, TA- and DNp73KO-mBOs was isolated at the indicated time points (see figure legends), using RNeasy mini Kit (Cat# 74106, QIAGEN, Hilden, Germany). RNA concentration and quality were determined using a Nanodrop ND-100 (Thermo Fisher scientific, Waltham, MA, United States). For each genotype and timepoint, RNA samples from three biological replicates were sent to Novogene Company Limited (Cambridge, England, United Kingdom) for RNA-sequencing (RNA-seq). The library preparations were sequenced on an Illumina platform (Illumina Inc., CA, United States) generating paired-end reads with 40 million reads per direction. The quality of sequencing was validated with FASTQC software (Simon, 2010). The alignment and quantification of the reads on the reference sequences were performed using Salmon software (Patro et al., 2017). The differential expression analysis was conducted using the R statistical analysis program, with the DESeq2 package (Love et al., 2014). Mapping of the reads to the reference genome, normalization to obtain the FPKMs (fragments per kilobase of transcript per million mapped reads) and DESeq2 analysis were performed at the core service Bioinformática USAL (Universidad de Salamanca, Spain). The FPKM raw data is available at OPEN SCAYLE: https://open.scayle.es/dataset/alonso-olivares-et-al-2024. Functional annotation analysis on differentially expressed genes (DEGs) was carried out using The Database for Annotation, Visualization and Integrated Discovery (DAVID) (Huang da et al., 2009; Sherman et al., 2022). Principal component analysis (PCA) was performed using Clustvis web tool (Metsalu and Vilo, 2015). Venn diagrams were created using BioVenn (Hulsen et al., 2008).

### 2.7 Gene expression analysis by reverse transcription and quantitative PCR (qRT-PCR)

cDNA synthesis from RNA samples was carried out using the High Capacity RNA-to-cDNA kit (Cat# 4387406, Applied Biosystems, Waltham, MA, United States). Gene expression was analyzed by qRT-PCR using FastStart Universal SYBR Green Master (Cat# 4913850001, Roche, Basel, CH) in a StepOnePlusTM Real-Time PCR System (Applied Biosystems). Primer sequences for target genes used are included in Supplementary Table S1. The comparative threshold cycle method was used to quantify relative mRNA expression.

### 2.8 Statistical analysis

Statistical analyses were performed using GraphPad Prism software version 10.2.3 for Windows (GraphPad Software, Boston, MA, United States). Values were expressed as mean ± standard error of the mean (SEM). Data distribution was analyzed in all cases. When comparing two groups, an unpaired Student’s t-test was used. For comparisons between multiple groups, statistical differences were determined using either parametric one-way ANOVA followed by Tukey’s correction or nonparametric Kruskal–Wallis tests with Dunn’s correction. Differences were considered significant when p < 0.05. Comparisons with WT mBOs are indicated by asterisks (*p < 0.05, **p < 0.01, ***p < 0.001), whereas hash (#) denote comparisons with p73KO organoids and dagger (†) is used to show comparisons with p53KO. ns: non-significant.

## 3 Results

### 3.1 Optimization of a rapid and robust system to generate mBOs from mouse pluripotent stem cells (mPSCs)

**FIGURE 1:**
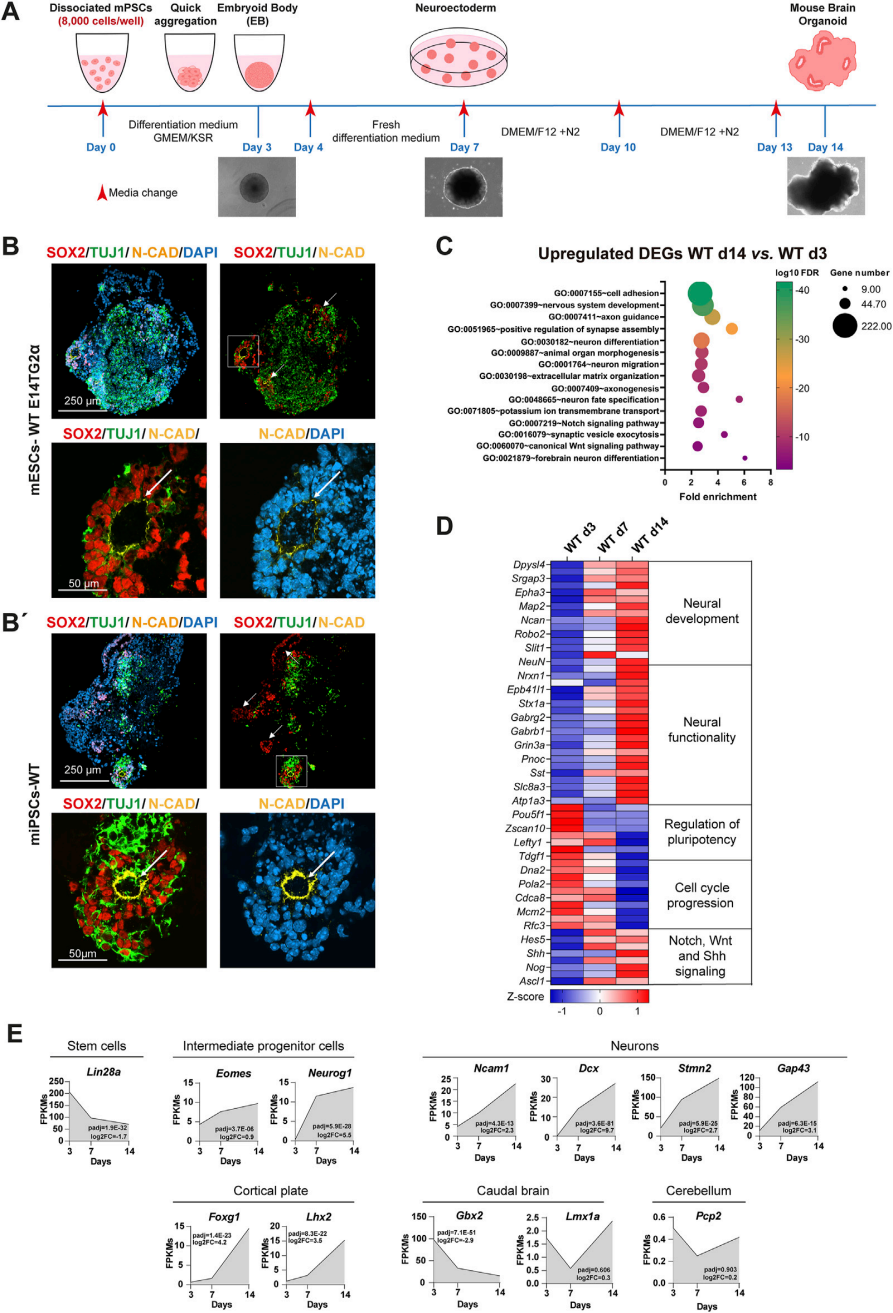
Optimization of a robust system to generate mBOs from mouse pluripotent stem cells. **(A)** Schematic overview of the optimized protocol for generating mBOs from mPSCs. **(B, B’)** Representative confocal images of mBOs immunostained for SOX2 (neural progenitor marker), TUJ1 (neuronal marker) and N-CAD (apical location in cells forming the ventricles) at day 14 of the differentiation process from the mESC line E14TG2α **(B)** and WT-iPSCs **(B’)**; white arrows indicate ventricle-like structures. The lower panels show a higher magnification image of the squared regions in the upper panels. Scale bars: upper panel: 250 μm; lower panel: 50 μm. **(C)** Functional annotation of DEGs upregulated between day 14 and day 3 WT-mBOs; FDR: false discovery rate. **(D)** Heatmap profiles showing RNA-seq expression Z-scores for representative gene set modules of DEGs among WT-mBOs at different time points. Representative gene names within the indicated functional module are shown. **(E)** Time course analysis of gene expression (FPKM values from RNA-seq) for genes related to the indicated categories: stem cells, intermediate progenitor cells, neurons, cortical plate, caudal brain and cerebellum.

Several protocols to generate mBOs have been described (Eiraku et al., 2008; Nasu et al., 2012; Watanabe et al., 2005), but they are not highly reproducible and require some adaptation to each cell line. We sought to establish an improved protocol to consistently generate high-quality, reproducible mBOs. We first used a previous method (Eiraku et al., 2008) to generate mBOs from WT-mPSCs, both mESC and miPSC lines. With this protocol, some mESCs differentiated into the neural lineage and gave rise to TUJ1+ neurons, but neural progenitors (SOX2+) failed to self-organize and form N-CAD+ ventricle-like structures (Supplementary Figure S1). We then adjusted some parameters to optimize the initial size of the organoids and improve cell viability: we increased the number of seeded cells from 3,000 to 8,000 cells/well and reduced the frequency of culture medium changing during the process (Figure 1A). We also modified the composition of the initial differentiation medium, eliminating Dickkopf Wnt Signaling Pathway Inhibitor 1 (DKK-1) and left-right determination factor 1 (LEFTY-1). DKK-1 inactivates Wnt/β-catenin signaling, which is necessary for NSC proliferation (Chenn and Walsh, 2002). On the other hand, LEFTY-1 inhibits Nodal signaling through pSMAD2/3 (Kim et al., 2014), so the presence of LEFTY-1 in the medium may prevent differentiation. Based on this reasoning, and in agreement with (Eiraku and Sasai, 2011), we eliminated both factors from the initial differentiation medium.

To determine whether the modified protocol could generate *bona fide* mBOs, we analyzed the expression and localization of key markers: SOX2 (a neural stem cell marker), TUJ1 (an early neuronal marker), and neural-cadherin N-CAD (a cell adhesion molecule). These markers are essential for assessing the presence and organization of VZ-like neural rosettes, a crucial indicator of proper brain organoid formation and development (Eichmuller and Knoblich, 2022). Indeed, rosette-like structures represent the *in vitro* counterpart of the neural tube, the structure of the early nervous system *in vivo* (Roll et al., 2022). We observed that WT mESC-derived mBOs contained SOX2+ neural progenitors at day 14, which were clearly organized in rosette-like structures surrounded by TUJ1+ neurons (Figure 1B). Rosettes were structurally similar to the VZ of the embryonic forebrain, with an internal lumen nicely decorated with N-CAD corresponding to the apical domain of neuroepithelial cells. This protocol was also efficient at generating mBOs from WT miPSCs (Figure 1B’), again with rosettes structurally similar to telencephalic VZ, thus confirming that the implemented modifications resulted in a robust protocol to generate mBOs from mPSCs.

To substantiate the quality of the mBOs generated with the improved protocol, we performed bulk transcriptomic analyses on mBOs derived from WT E14TG2α mESCs, analyzing them at different time points during development. Analysis of differentially expressed genes (DEGs) identified 3,083 genes that were significantly upregulated from day 3 to day 14 (padj <0.01; log2FC > 1), while 914 genes were downregulated (padj <0.01; log2FC < −1). Functional annotation analysis of the upregulated DEGs confirmed that our culture conditions induced transcriptional programs related to neurogenesis, highlighting Gene Ontology (GO) terms related to nervous system development, axon guidance, neuron fate specification, and forebrain neuron differentiation (Figure 1C). The transcriptomic data also showed progressive upregulation of pan neuronal markers such as *Map2, NeuN*, *Ncam1* and *Dcx*, as well as genes related to axon guidance (*Dpysl4 and Dpysl5, Robo2*) and neurite outgrowth (*Ncan*) during the process of mBO formation (Figure 1D; Supplementary Table S2). Upregulation of some of these DEGs associated with neuronal identity was further validated by qRT-PCR (Supplementary Figure S2). This upregulation was particularly striking in genes related to the organization of the cortical plate, such as *Foxg1* and *Lhx2*, whereas markers of the caudal brain (*Gbx2* and *Lmx1a*) or cerebellum (*Pcp2*) remained low (Figure 1E). Concomitantly, pluripotency genes such as *Pou5f1*, *Zscan10* or *Lin28A* were downregulated from day 3 to day 14 (Figures 1D, E; Supplementary Figure S2; Supplementary Table S2), together with genes associated with cell cycle progression and DNA synthesis, indicating a decrease in stem and proliferative cells. Some of the most prominent signaling pathways involved in neural induction and development include Notch, Sonic Hedgehog and Wnt (Manzari-Tavakoli et al., 2022). As expected, these signaling pathways were activated during the differentiation process and generation of mBOs, as shown by the upregulation of genes directly involved in signal transduction (*Shh* and *Notch1*), as well as some of their target genes (*Emx2*, *Ascl1* or *Neurogenin*), involved in neural development (Figures 1C–E).

Altogether, this confirmed that our optimized protocol promoted neural development and differentiation, leading to the generation and organization of mature cortical neurons from mPSCs. Therefore, we have fine-tuned and improved a protocol to generate mBOs by implementing changes in the initial cell number and the cocktail of factors used to induce neural commitment. This resulted in a rapid, affordable and highly reproducible protocol for the generation of mBOs from both induced and embryonic mPSCs.

### 3.2 p73 and p53 regulate neural progenitor proliferation and neuronal apoptosis in mBOs

**FIGURE 2:**
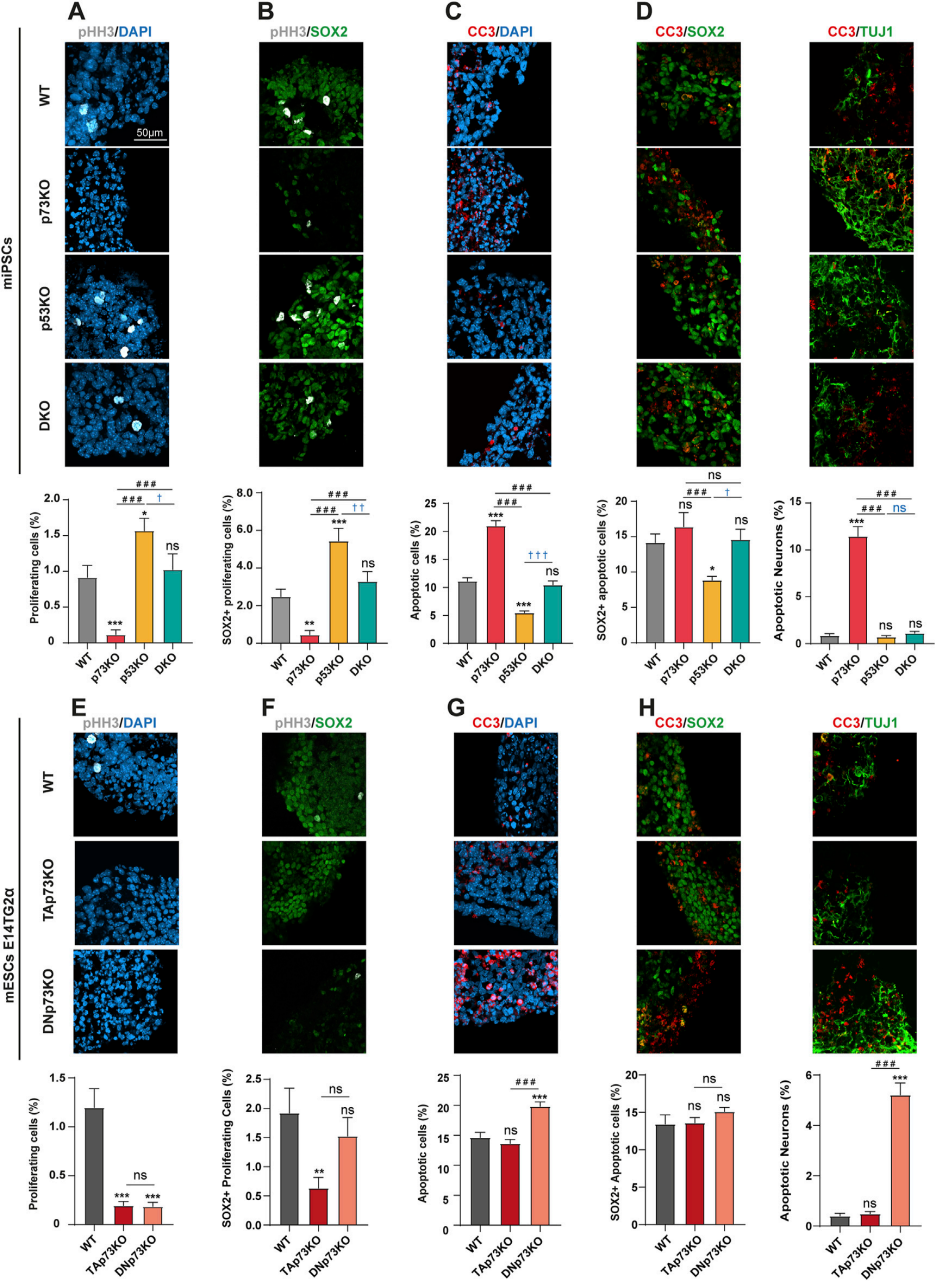
p73 and p53 regulate proliferation of neural progenitors and neuronal apoptosis within mBO in opposite but coordinated ways: TAp73 supports neural progenitor proliferation whereas DNp73 prevents p53-dependent neuronal apoptosis. All images are representative immunostaining confocal microscopy images of mBO cryosections at day 14 from miPSCs **(A–D)** or mESCs **(E–H)** with the indicated genotypes. **(A, E)** Immunostaining and quantification of proliferating cells (pHH3+). **(B, F)** Immunostaining and quantification of proliferating neural progenitors (pHH3+/SOX2+). **(C, G)** Immunostaining and quantification of apoptotic cells (CC3+). **(D, H)** Immunostaining and quantification of apoptotic neural progenitors (CC3+/SOX2+) or neurons (CC3+/TUJ1+). Scale bar: 50 μm. Data were collected from at least 30 mBOs from 2 different miPSC-clones or 3 different mESC-clones across 3 independent experiments and are represented as mean ± SEM. Statistical differences using Kruskal–Wallis test are indicated as: *, #, †, p-value <0.05; **, ##, ††, p-value <0.01; ***, ###, †††, p-value <0.001.

p53 and p73 are known cell cycle and cell death regulators during brain development (Agostini et al., 2018). Thus, we decided to corroborate these facts in the mBO model and to exploit our improved protocol to delve into the role of p53 and p73 in several aspects of mouse cortical brain development. We used previously generated p73KO-miPSCs, together with p53KO- and DKO-miPSCs (Martin-Lopez et al., 2017). Additionally, we made use of isoform specific mESCs (TAp73KO- and DNp73KO-mESCs) generated by CRISPR/Cas9 genome editing (Lopez-Ferreras et al., 2021). First, we assessed the capacity of these mBOs to recapitulate some of the effects of p53 and p73 deficiency on neural cell proliferation and/or cell death described *in vivo* (Gonzalez-Cano et al., 2010; Meletis et al., 2006). Similarly to what was observed in neurospheres assays (Meletis et al., 2006; Armesilla-Diaz et al., 2009), histological analysis of p53KO-mBOs after 14 days in differentiation conditions revealed a significant increase in the percentage of proliferative cells (positive for the mitosis marker pHH3) compared to controls (WT-mBOs) (Figure 2A). On the contrary, the absence of p73 led to a significant reduction in the percentage of proliferating cells. Interestingly, elimination of p73 in the context of p53-deficiency (DKO-mBOs), restored the proliferation index to levels close to WT-mBOs, suggesting that p73 is essential to sustain the high proliferation index unleashed by the absence of p53 within the mBO (Figure 2A).

To identify if the reduced proliferation observed in p73KO-mBOs affected their overall cellular populations, or if it was restricted to a specific cell type, we quantified the percentage of proliferating SOX2+ NPCs (pHH3+/SOX2+, Figure 2B). In WT-mBOs, the percentage of proliferating NPCs was around 2%, while this percentage drastically decreased to 0.45% in p73KO-mBOs (Figure 2B). Again, p53KO-mBOs doubled the percentage of SOX2+ proliferating cells, while DKO-mBOs had NPC proliferation rates comparable to their WT counterparts (Figure 2B). It is important to keep in mind that the decrease of proliferating cells in the p73KO could be related to a compensatory p53-activation leading the NPCs to exit the cell cycle, to senesce or to die.

Next, we analyzed apoptosis by quantification of CC3+ cells. In WT-mBOs, nearly 10% of cells were CC3+ (Figure 2C). Interestingly, while the absence of p53 in p53KO-mBOs significantly reduced, but not obliterated, this basal level of apoptosis, lack of p73 increased significantly the percentage of apoptotic cells in p73KO-mBOs compared to the WT-mBOs (Figure 2C). This effect was reduced to WT levels upon p53 loss in DKO-mBOs (Figure 2C), confirming a role of p73 in preventing p53-dependent cell death. However, it is noteworthy that a significant percentage of apoptotic cell death remains in double mutants, compared to mBOs lacking only p53, suggesting that p73 deficiency elicits a certain level of p53-independent apoptosis.

To determine whether p73 function is necessary to prevent overall apoptosis within mBOs or if its role is specific to a particular cell type, we performed double staining for SOX2/CC3 and TUJ1/CC3. As shown in Figure 2D, the lack of p73 did not significantly affect the frequency of apoptotic NPCs (SOX2+/CC3+ cells) but led to a sharp increase in the number of apoptotic neurons (TUJ1+/CC3+ cells).

Next, we sought to discriminate which p73 isoform was responsible for maintaining the proliferative pool of NPCs by analyzing cell proliferation and apoptosis in the p73 isoform-specific knockout-mBOs. In WT-mBOs, over 1% of the cells were pHH3+, a percentage severely reduced in mBOs lacking either TA- or DNp73 (Figure 2E), indicating that both isoforms are required to maintain the mitotic rate within the mBOs. Intriguingly, TAp73 deficiency led to a significant decrease in the number of SOX2+ NPCs, while DNp73 deficiency did not have a significant effect (Figure 2F). Together, these results indicate that TAp73 is the isoform necessary to sustain proliferation and maintain the pool of NPCs, whereas DNp73 is dispensable for the latter function, but essential for sustaining the proliferative capacity of other cell types in the mBO. The analysis of CC3+ cells revealed that while the loss of TAp73 had no effect on the overall apoptosis rate compared to WT-mBOs, DNp73-deficiency significantly increased the number of apoptotic cells (Figure 2G). Moreover, the lack of DNp73 primarily affected early immature neurons, but not SOX2+ progenitors (Figure 2H, right and left panels, respectively).

Collectively, our mBO model confirmed that p73 plays a key role in neuronal survival *in vitro*, consistent with previous observations *in vivo* (Tissir et al., 2009), where DNp73 plays a pro-survival role in discrete neuron types in a p53-dependent manner (Tissir et al., 2009; Osterburg and Dotsch, 2022; Pozniak et al., 2002; Pozniak et al., 2000). Moreover, our results evidenced that both TA- and DNp73 are necessary to maintain a functional pool of NPCs within the mBO, but they operate differently depending on the cell type: TAp73 maintains the proliferating NPC pool, while DNp73 sustains general proliferation and prevents neuronal apoptosis.

### 3.3 p53 and p73 are required for the self-assembly of mBOs

**FIGURE 3:**
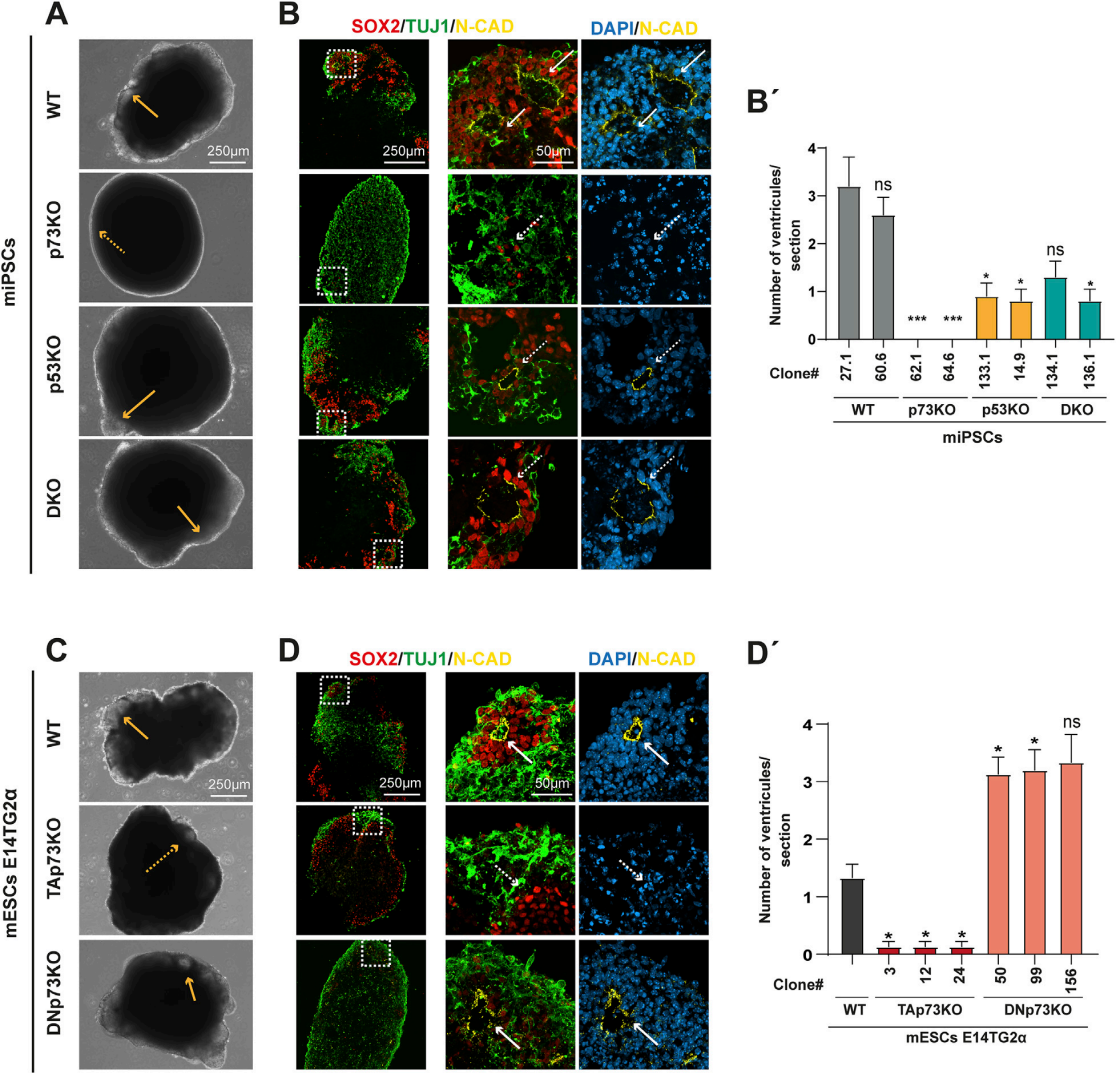
TAp73 is necessary for the correct organization of neural progenitors into ventricle-like structures, while DNp73 is dispensable. **(A, C)** Phase contrast images of mBOs derived from different miPSCs **(A)** or mESCs **(C)** genotypes. Orange arrows indicate buds with transparent lumens resembling neuroepithelium. Scale bar: 250 μm. **(B, D)** Representative confocal images of mBOs immunostained for SOX2 (neural progenitor marker), TUJ1 (neuronal marker) and N-CAD (apical location in cells forming the ventricles) at day 14 of the differentiation process. Dashed-squared areas on the left panels are magnified on the right panels; white arrows indicate well-formed ventricular structures; white dashed arrows indicate the absence of defined ventricular structures. Scale bars: 250 μm (left panel) and 50 μm (right panel). **(B’, D’)** Quantification of the number of ventricles *per* cryosection in different mBOs. The number of ventricles *per* cryosection is represented as mean ± SEM. Data were collected from at least 20 mBOs of the indicated clones from 3 independent experiments. Statistical differences using Kruskal-Wallis test are represented as: * p-value <0.05; *** p-value <0.001.

We next examined the effect of total loss of p73 and/or p53, as well as the elimination of the specific p73 isoforms, in the cellular and molecular events of mouse brain morphogenesis, beyond the already described impact in cell proliferation and apoptosis. mBOs at day 14 displayed obvious morphological differences between the four genotypes (Figure 3A). WT-mBOs exhibited multiple buds with translucent lumens resembling neuroepithelia (Figure 3A, yellow arrows) whereas the lack of p73, p53 or both (DKO) resulted in mBOs with less visible neuroepithelial structures, especially in the case of p73KO-mBOs which had almost smooth borders (Figure 3A, dashed arrows). This suggested that deficiencies in these p53 family members interfere with the correct development and morphogenesis of mBOs. A more detailed analysis of these epithelia-like buds revealed that WT-mBOs exhibited rosette-like structures formed by SOX2+ NPCs with an N-CAD+ ventricular surface (Figure 3B, arrows), surrounded by TUJ1+ neurons on the organoid surface, resembling the cortical plate in developing embryos (Roll et al., 2022). In contrast, the existing ventricles in mBOs lacking p73, p53, or both, were ill-defined, with an overall disrupted pattern (Figure 3B, dashed arrows). This was particularly striking in p73KO-mBOs, where neither N-CAD expression, nor defined ventricles, were observed (dashed arrows). Accordingly, while WT-mBOs presented an average of three defined neural ventricles *per* organoid cryosection, the mutant mBOs, and the p73KO-mBOs in particular, displayed a reduced number of ventricles (Figure 3B’). These observations suggest defects in cell-cell adhesion and cellular self-assembly, highlighting the essential requirement of *Trp73* for proper NPC organization and self-assembly into defined VZ-like structures.

### 3.4 TAp73 is necessary for the organization of NPCs into VZ-like structures

Having identified that *Trp73* is fundamental for mBO organization and the formation of VZ-like structures, we aimed to disclose the specific roles of the different p73 isoforms in this process. While WT- and DNp73KO-mBOs presented translucent neuroepithelial buds (Figure 3C, yellow arrows), mBOs lacking TAp73 were opaque, indicating defective formation of optically translucent and radially organized neuroectoderm. Besides, the neuroepithelial structures were less numerous and conspicuous in TAp73KO-mBOs (Figure 3C, dashed arrows).

Immunofluorescence analysis of mBO cryosections showed that mBOs generated from WT-mESCs exhibited NPCs organized into spherical rosettes with defined N-CAD+ ventricles, similar to the ones generated from WT miPSCs (Figure 3D, arrows). In contrast, NPCs in TAp73KO-mBOs were rather scattered and loose and did not form neural rosettes nor N-CAD+ ventricles (Figure 3D, dashed arrows), suggesting defects in NPC cell-cell adhesion, polarization and self-organization. In contrast, DNp73KO-mBOs showed well defined ventricles (Figure 3D, arrows), which were even more numerous than in WT-mBOs (Figure 3D’). This increased abundance of rosettes might be due to elevated TAp73 levels detected in these cells (Lopez-Ferreras et al., 2021), which could enhance their ability to establish correct cell polarity and form cell-to-cell interactions. In this differentiation context, this may favor the formation of VZ-like structures.

Considering all the above, the defective organization observed in p73KO-mBOs appears to be primarily due to the absence of the TAp73 isoform, seemingly essential for the organization of NPCs into rosette-like structures, and hence for the proper formation of ventricles within the mBO.

### 3.5 p73 isoforms, particularly DNp73, play a prominent role in the differentiation of NPCs

**FIGURE 4:**
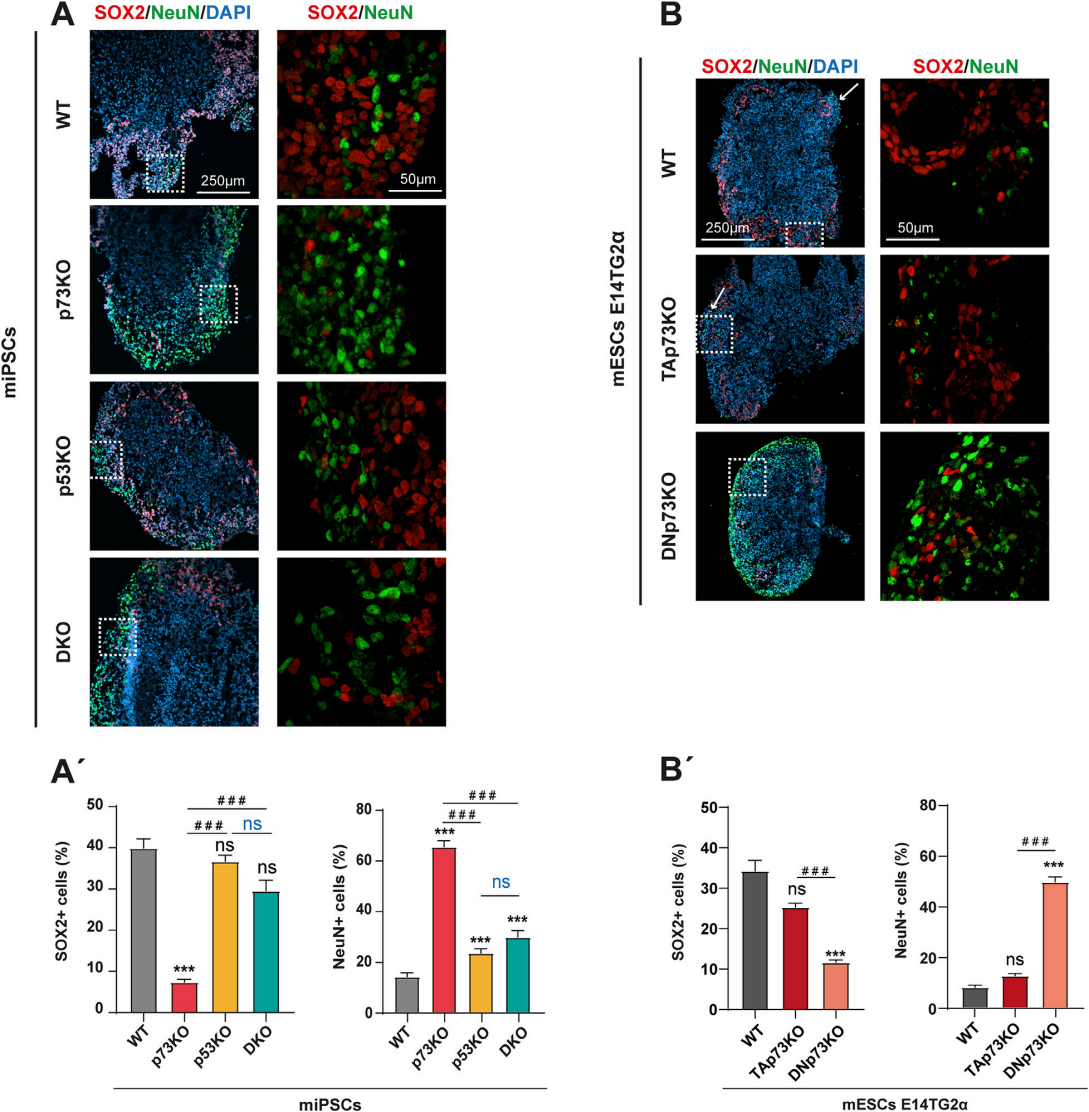
DNp73 absence leads to a depletion in the pool of neuronal precursors with a concomitant premature neuronal differentiation in a p53-dependent manner **(A, A’)** Representative confocal images **(A)** and quantification **(A’)** of mBOs generated from miPSCs at day 14, immunostained for SOX2 (neural progenitor marker), and NeuN (marker of postmitotic neurons). Dashed-squared areas on the left panel are magnified on the right panel. **(B, B’)** Representative confocal images **(B)** and quantification **(B’)** of mBOs generated from mESCs at day 14, immunostained for SOX2 (neural progenitor marker), and NeuN (marker of postmitotic neurons). Dashed-squared areas on the left panel are magnified on the right panel. Scale bars: 250 μm (left panel) and 50 μm (right panel). For the bar graphs in **(A’)** and **(B’)**, data were collected from at least 30 mBOs of 2 different miPSC-clones or 20 mBOs from 3 different mESC-clones across 3 independent experiments and are represented as mean ± SEM. Statistical differences using Kruskal–Wallis test are represented as: ***, ### p-value <0.001.

In the course of brain morphogenesis, NSCs differentiate to progressively acquire a specific neuronal identity and to eventually establish the complex structures of the brain. The processes of fate commitment and spatial organization are tightly regulated and, in fact, closely coupled. Thus, we explored whether the observed defects in neural structure organization within the mBOs were related to irregularities in the process of cellular decision-making and cell fate determination. In a typical differentiation process, SOX2+ progenitors give rise to TUJ1+ neurons which further differentiate into mature, post-mitotic neurons expressing the nuclear marker NeuN. Hence, we analyzed the expression of SOX2 and NeuN in mBOs at day 14 by immunofluorescence (Figure 4). In WT-mBOs, nearly 40% of cells were SOX2+ neural progenitors and 14% were NeuN+ neurons (Figures 4A, A’). The number of SOX2+ progenitors in mBOs lacking p53 was not significantly affected, while they exhibited a minor, but significant, increase (up to 23%) in the percentage of NeuN+ neurons (Figure 4A’), indicating that lack of p53 slightly affected the differentiation programs involved in neuronal maturation rather than the generation of neurons from SOX2+ progenitors. This is in agreement with previous studies highlighting the role of p53 in neuronal differentiation and maturation (Armesilla-Diaz et al., 2009; Forsberg et al., 2013; Marin Navarro et al., 2020). In contrast, mBOs lacking p73 showed significantly fewer SOX2+ neural progenitors and a noticeable significant increase in mature NeuN + cells (from 14% to 65%) (Figure 4A’). This indicates that the absence of p73 is associated with deregulation of neural cell fate, leading to a premature neuronal differentiation in these organoids, supporting previous data obtained using neurosphere assays (Fujitani et al., 2010; Gonzalez-Cano et al., 2010). Interestingly, the elimination of p53 in this context (DKO), decreased significantly, but not completely, this effect, suggesting a functional interaction between p73 and p53 in regulating cell fate and neural differentiation.

When analyzing the contribution of each p73 isoform, we observed that TAp73 deficiency did not significantly affect the number of SOX2+ progenitors nor the percentage of NeuN + neurons compared to WT-mBO (Figures 4B, B’). In contrast, the absence of DNp73 led to a notable decrease in the number of SOX2+ progenitors, accompanied by a highly significant increase in the number of mature neurons (from 8% in WT-mBOs to 50% in DNp73-mBOs). In DNp73-mBOs, these neurons were detected throughout the organoid rather than mainly in the cortical border, as was the case in WT- and TAp73KO-mBOs (Figure 4B, white arrows). This is in line with the phenotype observed in p73KO-mBOs and confirms that accelerated neuronal differentiation in these organoids is caused by the absence of DNp73. These results demonstrate that DNp73 has a remarkable effect in orchestrating neuronal differentiation from neural progenitors and may specifically act as a negative regulator of neuronal cell fate.

### 3.6 TAp73 and DNp73 exert their function through the regulation of distinct transcriptional programs

To further pinpoint the transcriptional changes occurring in the p73 isoform-deficient mBOs, we conducted global transcriptomics analyses using bulk RNA sequencing at days 7 and 14 (Supplementary Table S3). PCA analysis showed that clustering of biological replicates from WT, TA- and DN-p73KO organoids improved over time, with a clear separation between days 7 and 14 (Supplementary Figure S3A). Moreover, the major source of variation was linked to the mBOs genotype (with principal component PC1 accounting for almost 40% of the variance). The specific loss of either TA- or DN-p73 resulted in altered transcriptional profiles (Supplementary Figure S3B), with the effect being more pronounced at later stages. Moreover, DNp73 deficiency had a greater impact on the number of DEGs, particularly at day 14: 2,835 genes were upregulated (padj <0.01; log2FC > 1) and 2,350 genes became downregulated (padj <0.01; log2FC < −1) compared to WT-mBOs.

**FIGURE 5:**
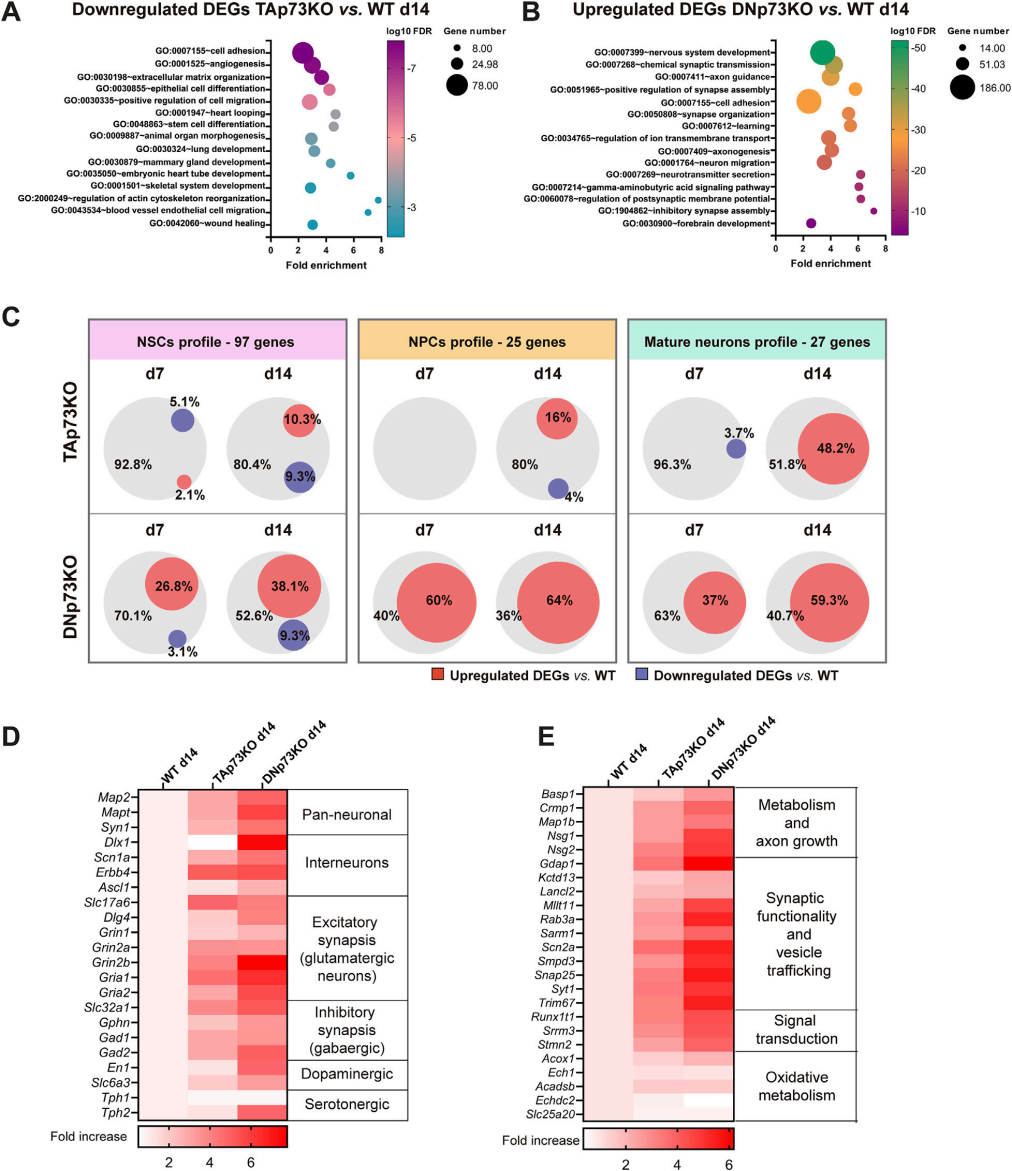
TA- and DNp73 regulate distinct transcriptional programs related to brain development and nervous system function. **(A, B)** DEGs with a p-adj <0.05 were used for GO analysis using DAVID Software. **(A)** Functional annotation of downregulated DEGs in TAp73KO-mBOs compared to WT after 14 days in culture. **(B)** Functional annotation clustering of upregulated DEGs in DNp73KO-mBO compared to WT after 14 days in culture. **(C)** Venn diagrams illustrating the percentage of transcripts related to neural stem cells (NSCs), neural progenitor cells (NPCs) and mature neuron profiles which are upregulated (red) or downregulated (purple) in TAp73KO or DNp73KO-mBOs at day 14. **(D)** Heatmaps of DEGs related to different neuronal identities in TAp73 and DNp73KO-mBOs at day 14. **(E)** Heatmaps of DEGs related to metabolism, axon growth, synaptic functionality, signal transduction and oxidative metabolism in TAp73KO and DNp73KO-mBOs at day 14.

TAp73 is a *bona fide* transcriptional activator, while DNp73 acts mostly, but not always (Toh et al., 2008), as a repressor. Thus, we focused on genes that were downregulated in the absence of TAp73 and upregulated upon DNp73 deficiency. Functional annotation analyses of the DEG lists revealed that downregulated genes in TAp73KO-mBOs were associated with cell adhesion, extracellular matrix organization, animal organ morphogenesis (e.g., heart tube development, mammary gland development, etc.) and axis specification (Figure 5A). This reinforces the role of TAp73 in regulating a transcriptional module related to tissue morphogenesis (Maeso-Alonso et al., 2021) and may explain the observed inability to form archetypal structures, such as proper VZ-like structures, in the absence of TAp73. Notably, genes associated with functions previously linked to TAp73, such as angiogenesis, stem cell differentiation, and cytoskeleton regulation (Gonzalez-Cano et al., 2010; Fernandez-Alonso et al., 2015; Fuertes-Alvarez et al., 2018), were also enriched. On the other hand, upregulated genes in DNp73KO-mBOs were highly significantly associated to biological terms such as nervous system development, synaptic transmission, axonogenesis or axon guidance (Figure 5B), correlating with the phenotype of premature neuronal differentiation observed in DNp73KO-mBOs. Additionally, GO terms linked to DNA replication, cell cycle progression, cell division and stem cell population maintenance were enriched among downregulated DEGs in DNp73KO-mBOs (Supplementary Figure S3C), indicating that DNp73 sustains the capacity of NPCs to proliferate.

To evaluate how the elimination of TA- or DN-p73 affected neural cell fate determination, we intersected the up- and downregulated DEGs at different stages (compared to their WT counterparts), with gene sets that have been previously linked to NSCs, NPCs and mature neurons (Bifari et al., 2020; Cahoy et al., 2008; Ciarpella et al., 2021). As shown in Figure 5C, none of the genes related to the NPCs- or the mature neurons-profiles were downregulated in DNp73KO-mBOs, and only a few were repressed in TAp73KO. It called our attention that three NSC-associated genes were commonly downregulated in DNp73KO and TAp73KO-mBOs both at day 7 and 14: NADPH-Dependent Carbonyl Reductase 3 (*Cbr3*), Thiosulfate Sulfurtransferase (*Tst*) and ETS Variant Transcription Factor 4 (*Etv4*). The downregulation of *Etv4* is of particular interest, since it works as a mechanical transducer that drives spatiotemporal lineage specification and its inactivation in human embryonic stem cell epithelia derepresses the neuroectoderm fate (Yang et al., 2024). On the other hand, DNp73KO-mBOs remarkably upregulated genes related to the neural fate when compared to the WT counterparts, whereas the lack of TAp73 had a milder outcome. Indeed, elimination of TAp73 had almost no impact at early stages (day 7) (Figure 5C). On the contrary, the effect in DNp73KO-mBOs was striking at early stages, with DNp73 deficiency resulting in the upregulation of 60% of the genes included in the NPC profile and a 37% overlap with genes related to mature neurons at day 7. This suggests that DNp73 absence accelerates neuronal lineage induction.

The importance of DNp73 repression, and TAp73 to a minor extent, in establishing neuronal identity is further illustrated by the significant upregulation of individual markers related to various neuron types (Figure 5D). For instance, isoform-deficient mBOs upregulate genes that have been shown to be important for the function of interneurons, such as *Dlx1*, *Scn1a* or *Erbb4* (Ciarpella et al., 2021). In addition, DNp73KO-mBOs robustly overexpress genes related to excitatory and inhibitory synapses. The enrichment in glutamatergic synapses was evidenced, for example, by the upregulation of the glutamate transporter *Slc17a6* (VGLUT2), as well as the overexpression of NMDA and AMPA receptors (*Grin2b*, and *Gria1a/Gria2*, respectively). On the other hand, the presence of GABAergic synapses was indirectly assessed by the increased expression of GABA transporter (*Slc32a1*) and gephyrin (*Gphn*). Conversely, markers indicative of other neuronal lineages (e.g., serotonergic) were not consistently expressed. Collectively, our results indicate the presence of a diversely enriched population of neurons in p73 deficient organoids. Finally, we examined a previously described gene set of mature neuron markers (Ciarpella et al., 2021). The expression levels of genes related to: i) metabolism and axon growth, ii) synaptic functionality and vesicle trafficking and iii) signal transduction and neurotransmission regulation were increased in both genotypes, most notably in DNp73KO-mBOs, indicating an enhanced neuronal maturation (Figure 5E). However, genes related to beta-oxidation pathways at peroxisomal (*Acox1, Ech1*) or mitochondrial level (*Acadsb, Echdc2, and Slc25a20*) (Watanabe et al., 2007) were not significantly upregulated, suggesting that at the latest analyzed time point the metabolic switch to oxidative metabolism did not accompany the neuronal maturation observed in those organoids.

**FIGURE 6:**
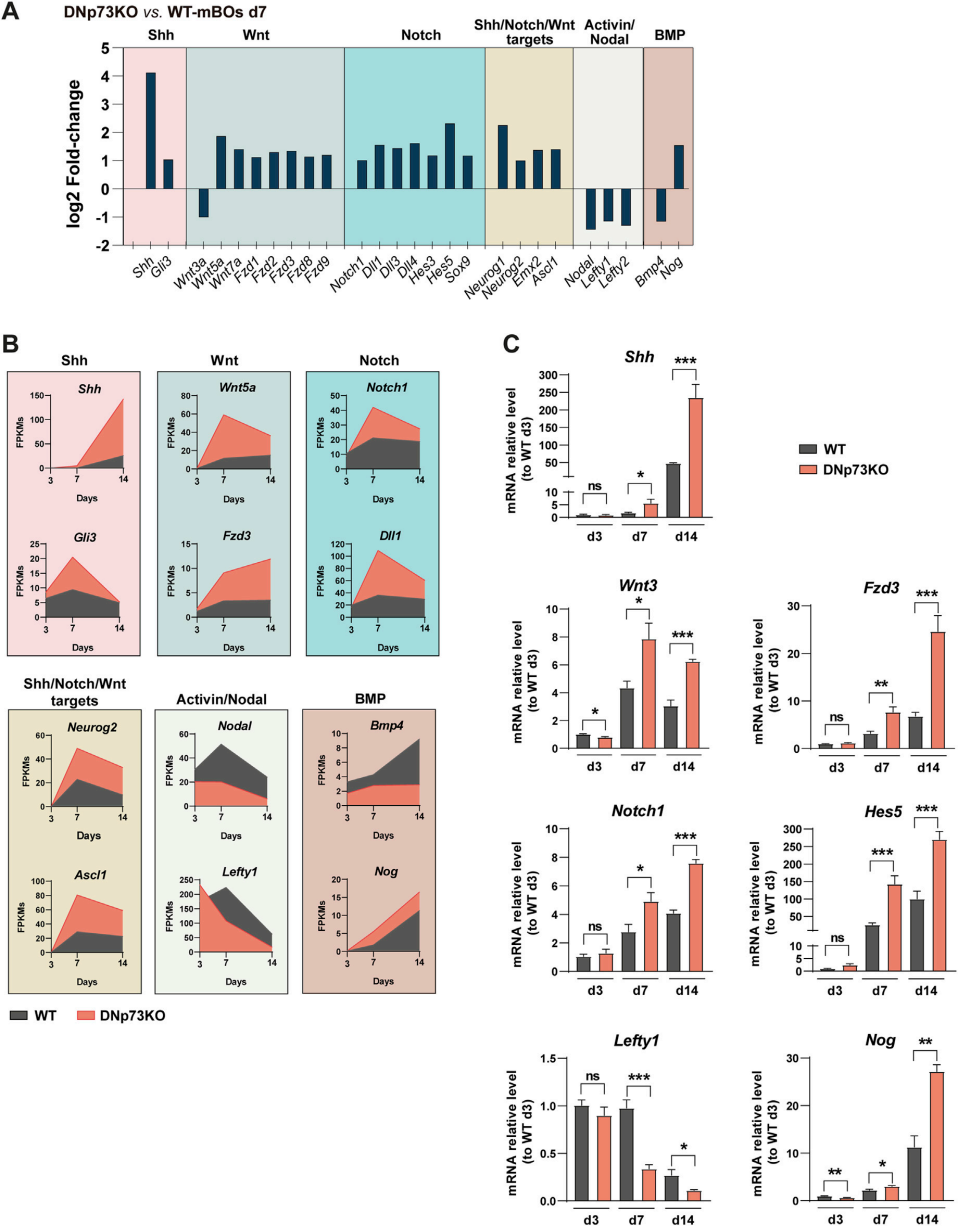
Lack of DNp73 results in the enhanced activation of the Shh, Wnt, and Notch signaling pathways, along with an early and strong repression of BMP and Activin/Nodal signaling. **(A)** Bar plot showing the expression of a series of transcripts corresponding to the indicated signaling pathways. The log2 Fold Change of DNp73 mBO at day 14 compared to WT mBOs at day 14 is shown. **(B)** Time course analysis of gene expression corresponding to genes related to the indicated signaling pathways. **(C)** Expression levels of the indicated transcripts were analyzed in WT and DNp73KO-mBOs at days 3, 7, and 14 of the optimized protocol using quantitative PCR (qPCR). The data represent the mean ± SEM of four replicates, derived from two independent experiments. Statistical significance was assessed using Student’s t-test and is denoted as follows: * p < 0.05; ** p < 0.01; *** p < 0.001.

Brain cortex development is a highly orchestrated sequence of events that requires precise signaling inputs. Shh signaling is vital for the formation and maturation of cortical layers, as it regulates the timing of progenitor cell differentiation and promotes their proliferation (Cai et al., 2023; Saade et al., 2017). These processes affect the thickness and cell composition of each cortical layer (Belmonte-Mateos and Pujades, 2021; Nowakowski et al., 2017). The Notch signaling pathway is pivotal in maintaining NPC populations and regulating their differentiation into specialized neurons and glia (Gaiano and Fishell, 2002), thereby influencing the overall size and complexity of the cortical region within the organoid. Additionally, the timing of Wnt activation is particularly crucial for establishing neural identity and suppressing unwanted cell fates (Rosso and Inestrosa, 2013), ensuring that cortical brain organoids develop with the appropriate cellular composition and organization. Supporting the role of DNp73 in regulating neural cell fate, we observed significant overexpression of some key genes involved in these pathways in DNp73KO-mBOs at day 7 (Figure 6A). This was evidenced by the upregulation of key genes involved in the activation and transduction of these pathways (*Notch1, Shh, and Wnt5a*), as well as their target genes (*Emx2, Ascl-1*, and *Neurog1*) (Figures 6A, B). Additionally, the induction of the neural lineage requires dual SMAD inhibition (Chambers et al., 2009). In accordance, DNp73KO-mBOs also exhibited downregulation of *Bmp-4* and *Nodal*, which promote the activation of Smad1/5/8 and Smad2/3 signaling, respectively, suggesting that the absence of DNp73KO leads to dual-SMAD inhibition.

Gene expression dynamics in DNp73KO-mBOs from day 3 to day 14, as indicted by our transcriptomic data and validated by qRT-PCR analysis, reflected the activation of the Shh, Wnt, and Notch signaling pathways (Figures 6B, C). Regarding the Activin/Nodal signaling, it is known that this cascade regulates *Nodal* and *Lefty1* gene expression and, that while Lefty1 blocks Nodal signaling (Chen and Shen, 2004), *Lefty1* expression pattern follows essentially that of *Nodal* in early stages of development (Besser, 2004; Zhang et al., 2019). Thus, in agreement with a repression of this signaling cascade, we detected a significant reduction of *Lefty1* expression in DNp73KO-mBOs from days 7–14 (Figures 6B, C). This repression of Nodal signaling could account for the switch towards a predominant neuroectodermal fate observed in the absence of DNp73 at early stages (Figure 5C), as previously reported in ESCs (Vallier et al., 2004; Wang et al., 2017), pointing to DNp73 as a node regulator of neuronal fate. Altogether, these characteristic molecular signatures highlight the importance of p73, particularly DNp73, for a precise and timely regulation of the onset of neural cell fate commitment and shed new light on the specific role of each p73 isoform, and how they tightly coordinate the maintenance of a pool of proliferating neural progenitors with the timely generation of neurons, and their maturation.

## 4 Discussion

Generating cellular models that accurately reflect the complexity and uniqueness of the mammalian brain is a major challenge in cell biology. In this work, we refined a 3D *in vitro* system to generate mBOs that recapitulate the main structural and developmental characteristics of the mouse cerebral cortex. We introduced several modifications into the protocol previously defined by Eiraku and coworkers (Eiraku et al., 2008), aimed at optimizing the initial number of cells to constitute the aggregates and increase their survival. These changes resulted in a rapid, affordable and robust method that efficiently induced the differentiation of pluripotent stem cells, mESCs and miPSCs, into neural progenitors and neurons, which constituted distinctive 3D VZ-like neurogenic structures. As it has been suggested before (Eiraku et al., 2008; Watanabe et al., 2005; Eiraku and Sasai, 2011; Watanabe et al., 2007; Gaspard et al., 2008), the onset of neural differentiation is predominantly dependent on cell-autonomous mechanisms rather than on inductive signaling. This illustrates why the process of mBO generation is exquisitely influenced by minor changes in the initial cell density and basal culture conditions (Eiraku et al., 2008; Watanabe et al., 2005; Wataya et al., 2008).

Murine cerebral organoids have also been generated from NSC (Ciarpella et al., 2021). The protocol developed by Ciarpella and colleagues efficiently generates brain organoids composed of mature and functional neurons after 32 days in culture. Although these authors reported a very interesting tool for screening purposes, the fact that they used mouse embryos as a source of NSCs, instead of mESCs or miPSCs, makes the protocol more laborious, limits the throughput and still relies on the use of animals compared to ours. In addition, our protocol is shorter (14 days) and requires less exogenous factors, which determines a faster and cheaper production of neuron-bearing organoids that can be useful to study the early developmental stages of mouse brain development. Therefore, despite previous attempts of generating 3D forebrain structures from mPSCs (Eiraku et al., 2008) and more recent achievements in obtaining these structures from NSCs (Ciarpella et al., 2021), the protocol described here reports the efficient production of mBOs from PSCs in a fast, affordable and robust manner.

Applying our optimized protocol to analyze the function of p53 family members in mouse brain morphogenesis, we have been able not only to confirm some biological findings obtained from other *in vitro* and *in vivo* models (Agostini et al., 2010; Gonzalez-Cano et al., 2010; Talos et al., 2010), but also to provide a more comprehensive reading of the distinct roles played by p73 isoforms in this particular context. Our data showed that p73 deficiency results in a dramatic reduction in NPCs, likely due to a combination of premature neuronal differentiation and decreased proliferation capacity of neural progenitors. This supports a model in which the progenitor population fails to properly expand, exits the cell cycle prematurely, and initiates neuronal differentiation. Previous *in vivo* studies from our group have shown that p73 deficiency halts the transition of RGCs into ependymal cells (Gonzalez-Cano et al., 2016). RGCs function as neurogenic progenitors during brain development, and therefore, the previously defined role of p73 in RGCs is consistent with our *in vitro* findings regarding neural progenitor differentiation.

During development, neurogenic progenitors must polarize and establish cell-cell adhesions to organize in ventricular structures. Disruptions in these functions *in vivo* can lead to structural defects in the neurogenic niches, including the ependymal cell layer that lines and defines the VZ (Gonzalez-Cano et al., 2016). In addition, p73 has been implicated in regulating planar cell polarity (PCP) in different contexts, including the neurogenic niche of the SVZ of the brain (Gonzalez-Cano et al., 2016), as well as in the proper establishment of adherens and tight junctions in different cell types (Maeso-Alonso et al., 2021; Lopez-Ferreras et al., 2021; Holembowski et al., 2014). This endorses the results obtained in mBOs, in which we report that p73 deficiency hinders the formation of ventricular structures early during development.

The differential contribution of the p73 isoforms to the regulation of neurogenesis and brain development has been a longstanding question, as the phenotypes observed in the TA- and DN-specific knockout mice are intriguing. *Trp73*KO mice, that lack all p73 isoforms, are runty and exhibit very severe brain anomalies such as hydrocephalus and hippocampal dysgenesis (Yang et al., 2000). TAp73KO mice partially recapitulate this phenotype and present hippocampal dysgenesis too, whereas DNp73KO show a mild phenotype characterized by neurodegeneration (Tissir et al., 2009; Wilhelm et al., 2010). To distinguish the molecular function of the p73 isoforms, we took advantage of the mPSCs previously generated in our group, in which TA- and DNp73 isoforms were specifically ablated (Lopez-Ferreras et al., 2021), and generated mBOs. We found that TAp73 is essential for precise structural organization of neural progenitors. Also, our transcriptome analysis shows that TAp73 regulates a transcriptional module involved in cell adhesion, extracellular matrix organization and morphogenesis, which mechanistically underlies the establishment of the structures which are found disrupted in TAp73KO-mBOs. On the other hand, DNp73KO-mBOs exhibit premature neuronal differentiation, which seems counterintuitive with the reported role of DNp73 as a pivotal regulator of neuronal survival (Tissir et al., 2009). However, the neurons observed in mBOs are highly apoptotic, which is indeed consistent with the previously *in vivo* observation.

The increased apoptosis observed in p73KO-mBOs is compensated after p53 loss, indicating that neuronal apoptosis resulting from p73 elimination is p53-dependent. In mBOs, the absence of p53 led to a subtle disorganization, even in the absence of p73 (DKO-mBOs), as well as a slight accelerated neuronal differentiation, in accordance with previous data shown in human p53 knockdown organoids (Marin Navarro et al., 2020). All these results show that p53 family members play important and largely non-redundant roles in mouse brain development.

Mechanistically, the functional interaction between p73 isoforms and p53 regulation has been well studied. Like p53, TAp73 can transactivate target genes that regulate apoptosis and senescence (Rozenberg et al., 2021). On the other hand, DNp73 acts as a dominant negative for TAp73 (and p53) through a direct competition for the promoter or by formation of inactive hetero-oligomeric complexes, showing an antiapoptotic effect (Di et al., 2013). Moreover, both TAp73 and p53 activate the DNp73 promoter creating a negative feedback loop (Rozenberg et al., 2021). Thus, DNp73 is activated after DNA damage in a p53-dependent manner to regulate p53-induced cell cycle arrest (Vossio et al., 2002). However, the pro-survival role of DNp73 can only partially explain the neural phenotypes, both in the various p73 knockout models (Niklison-Chirou et al., 2017) and in our mBOs.

Based on our proliferation, apoptosis and transcriptomic data, we hypothesize that DNp73 is essential to sustain proliferating neural progenitor cells, but it is also a regulator of early neuronal fate acquisition during development. The precise patterning of brain organoids relies heavily on the strength and duration of exposure to diffusible morphogens such as Shh and Wnts, as well as the intricate crosstalk among different signaling pathways such as Notch (Zhao and Haddad, 2024). In our model, lack of DNp73 results in the enhanced activation of the Shh, Wnt, and Notch signaling cascades, along with an early and strong repression of BMP and Activin/Nodal signaling. The functional interplay of p73 isoforms with these signaling pathways, especially DNp73, is not yet completely understood. Several reports have linked p73 function to the Notch signaling pathway since the loss of total p73 leads to the transcriptional dysregulation of *NOTCH-1, NOTCH-2, HES-5, JAG2, HEY-2,* and *DELTEX* (Niklison-Chirou et al., 2016), being *HEY2* a direct TAp73 transcriptional target (Fujitani et al., 2010). Moreover, TAp73 isoforms, but not DNp73, are capable of directly binding the Notch-1 intracellular domain (N1ICD) and antagonize its transcriptional activity (Hooper et al., 2006). However, additional studies are required to unravel how DNp73 physiologically regulates these factors.

On the other hand, *Wnt3* and its receptor *Fzd1* are direct p53, TAp63 and TAp73 target genes in ESCs (Wang et al., 2017). Thus, the elevated TAp73 levels detected in DNp73KO cells (Lopez-Ferreras et al., 2021) could be responsible for the enhanced Wnt signaling. However, whether DNp73 acts directly over this signaling cascade remains to be clarified. The interaction between p73 and the BMP pathway is better understood. P73 is a positive modulator of the BMP circuit; DNp73 expression enhances BMP4-induced Smad1/5/8 activation and directly represses the activation of the *SMAD*6 promoter (Martin-Lopez et al., 2017). Thus, DNp73 depletion could hinder BMP signaling cascade during mBO formation. Moreover, similarly to our observation in mBOs, during ESC differentiation, depletion of p53 and p73 inhibited Smad2/3/Nodal signaling, and the p53/p73-depleted cells adopted a neuroectoderm fate (Wang et al., 2017). This change into an alternative neuroectoderm fate is consistent with Nodal inhibition during development (Vallier et al., 2004). Thus, our data support the idea of DNp73 as a regulator of the early neuroectoderm fate switch.

Overall, we have optimized a model to efficiently generate mouse cortical brain organoids. In fact, we have utilized this *in vitro* culture system to gain further insight into the specific function of p53 and p73 isoforms in brain morphogenesis, laying the foundation of a new understanding of p53 and p73 in neurodevelopment and validating the reliability of this 3D model as a useful tool for biomedical and neurodevelopmental biology research. Moreover, we propose a dual role of p73 regulating brain morphogenesis, whereby TAp73 governs transcriptional programs essential for the establishment of the neurogenic niche structure, and DNp73 is central for a precise and timely regulation of neural cell fate.

## Data Availability

The datasets presented in this study can be found in online repositories. The names of the repository/repositories and accession number(s) can be found below: https://open.scayle.es/dataset/alonso-olivares-et-al-2024, Alonso-olivares-et-al-2024.
