# Supplementary material for: Mouse cortical organoids reveal key functions of p73 isoforms: TAp73 governs the establishment of the archetypical ventricular-like zones while DNp73 is central in the regulation of neural cell fate
Source: Front Cell Dev Biol. 2024 Sep 23;12:1464932. doi: 10.3389/fcell.2024.1464932 (PMC11456701; doi:10.3389/fcell.2024.1464932)
Supplement: Supplementary file 1 [file DataSheet1.docx]

***Supplementary Material***

# Supplementary Data

# Supplementary Figures and Tables

For more information on Supplementary Material and for details on the different file types accepted, please see [here](https://www.frontiersin.org/guidelines/author-guidelines#supplementary-material).

## Supplementary Figures

**2.1.1 Supplementary Figure 1**


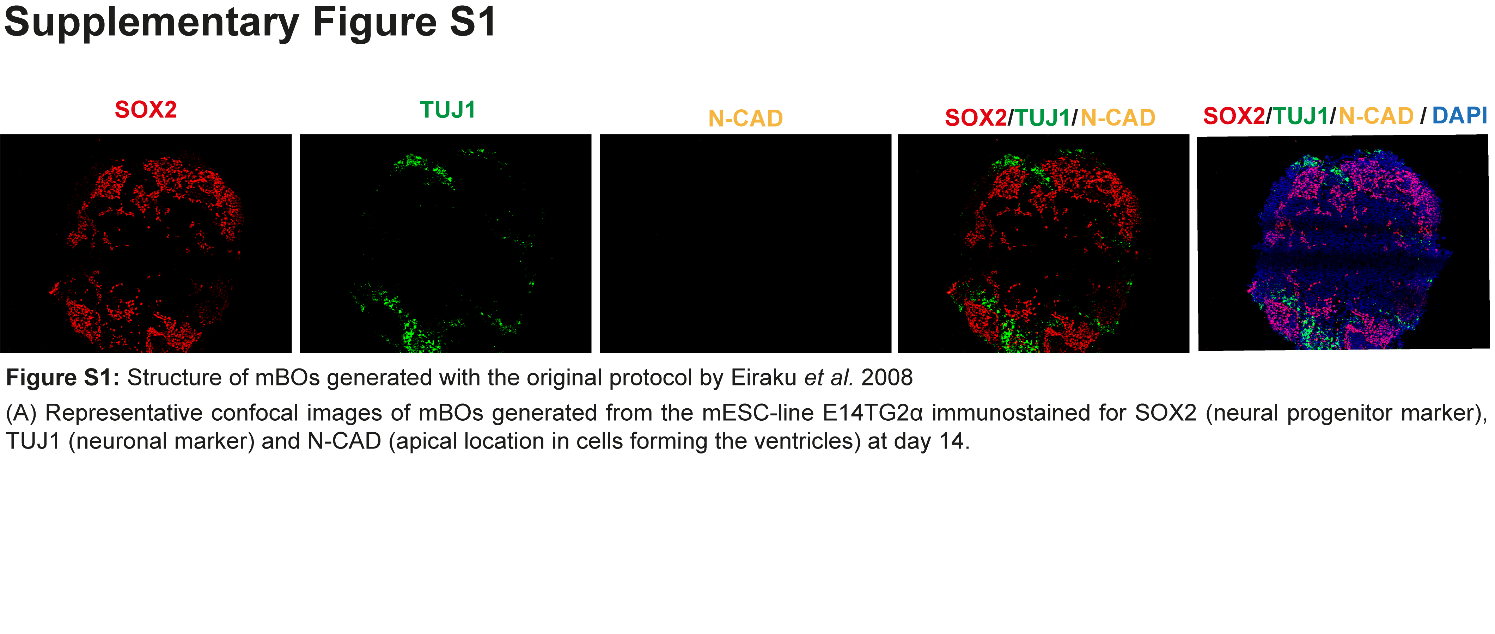


**2.1.2 Supplementary Figure 2**

**
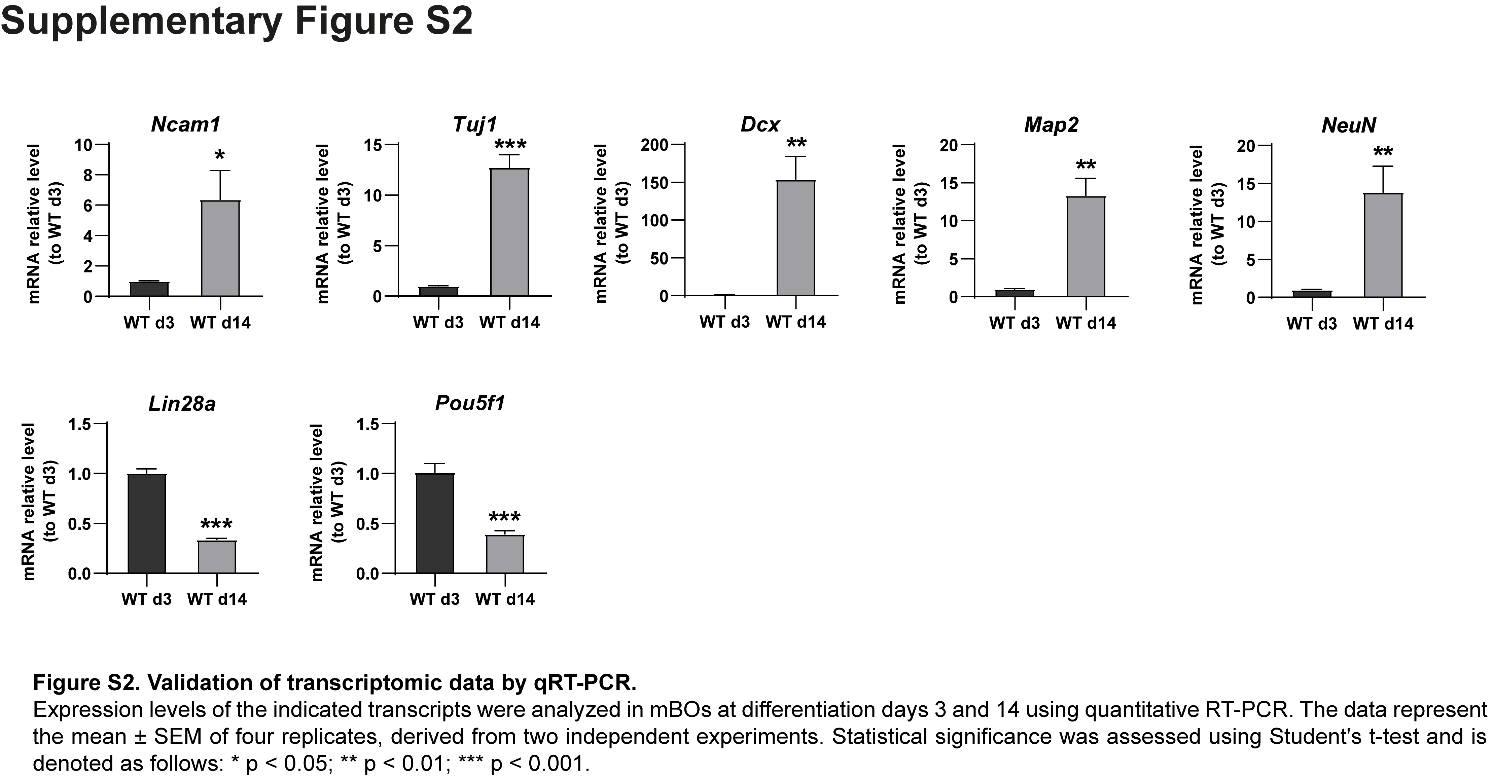
**

**2.1.3 Supplementary Figure 3**

**
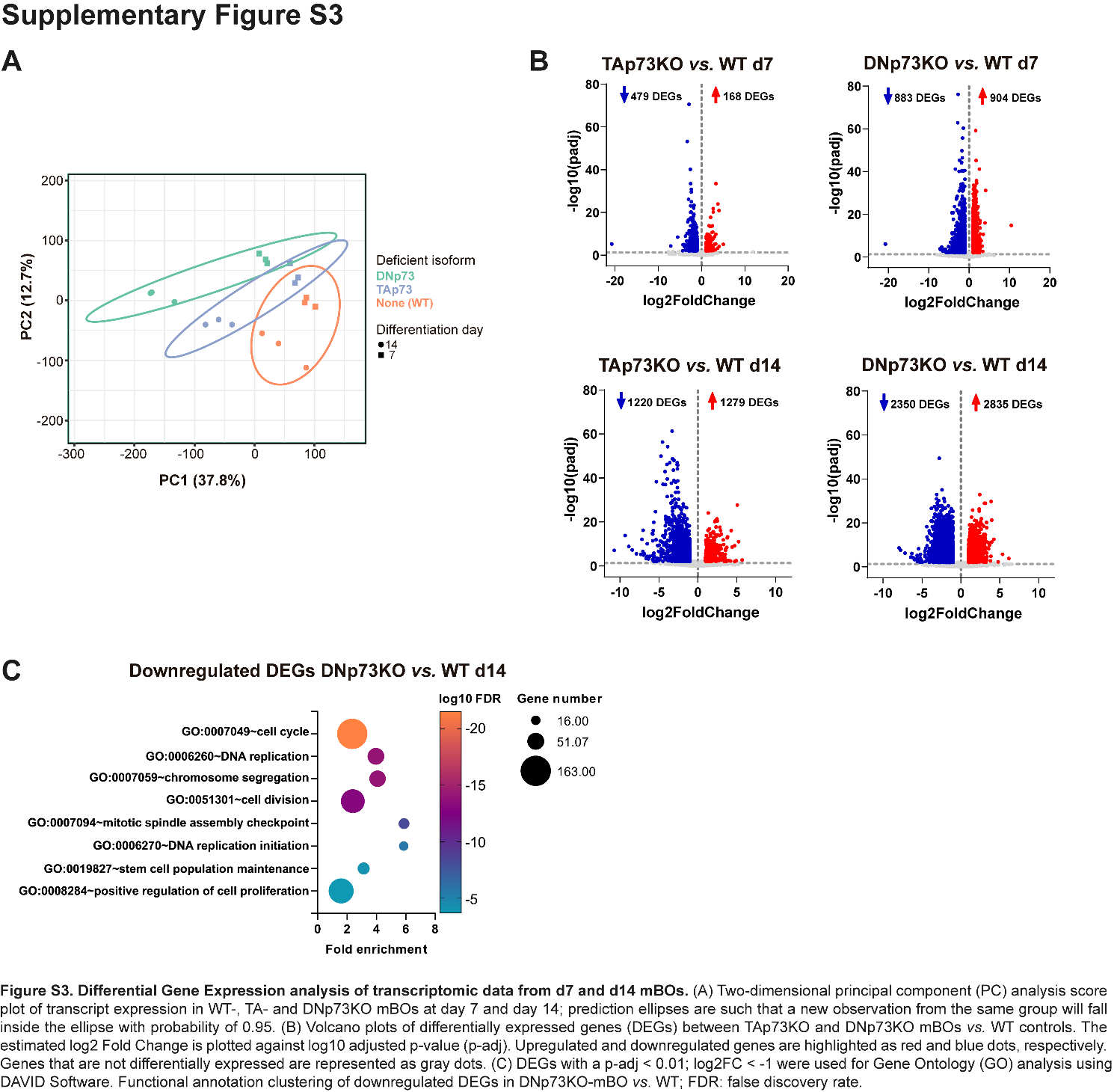
**

## Supplementary Tables

- - 1. **Supplementary Table 1. List of primers used for qRT-PCR**

| **Transcript** | **Forward (5´-3´)** | **Reverse (5´-3´)** |
| --- | --- | --- |
| Ncam1 | GACAGAACCCGAAAAGGGC | GTTGGGGACCGTCTTGACTT |
| NeuN | ATCGTAGAGGGACGGAAAATTGA | GTTCCCAGGCTTCTTATTGGTC |
| Dcx | CATTTTGACGAACGAGACAAAGC | TGGAAGTCCATTCATCCGTGA |
| Shh | AAAGCTGACCCCTTTAGCCTA | TTCGGAGTTTCTTGTGATCTTCC |
| Lefty1 | CCAACCGCACTGCCCTTAT | CGCGAAACGAACCAACTTGT |
| Notch 1 | GATGGCCTCAATGGGTACAAG | TCGTTGTTGTTGATGTCACAGT |
| Pou5f1 | ATGGCATACTGTGGACCTC | AGCAGCTTGGCAAACTGTTC |
| Lin28a | GGCATCTGTAAGTGGTTCAACG | CCCTCCTTGAGGCTTCGGA |
| Tuj1 | TAGACCCAGCGGCAACTAT | GTTCCAGGTTCCAAGTCCACC |
| Map2 | TCAGAGGCAATGACCTTACC | GTGGTAGGCTCTTGGTCTTT |
| Wnt3 | CTCGCTGGCTACCCAATTTG | CTTCACACCTTCTGCTACGCT |
| Fzd3 | ATGGCTGTGAGCTGGATTGTC | GGCACATCCTCAAGGTTATAGGT |
| Hes5 | AGTCCCAAGGAGAAAAACCGA | GCTGTGTTTCAGGTAGCTGAC |
| Noggin | GCCAGCACTATCTACACATCC | GCGTCTCGTTCAGATCCTTCTC |
| 18S (housekeeping) | AGTTCCAGCACATTTTGCGAG | TCATCCTCCGTGAGTTCTCCA |

### Supplementary Table 2. Average Z-score of RNA-seq expression values computed for genes in Figure 1 D

|  |  | **Average Z score** | | |
| --- | --- | --- | --- | --- |
|  | **Gene name** | **WT day 3** | **WT Day 7** | **WT Day 14** |
| **Neural development** | *Dpysl4* | -1.09009 | 0.44059 | 0.64950 |
|  | *Dpysl5* | -0.96372 | 0.17993 | 0.78379 |
|  | *Srgap3* | -1.16537 | 0.52881 | 0.63656 |
|  | *Ephb3* | -0.94378 | -0.17463 | 1.11842 |
|  | *Epha3* | -1.13524 | 0.82303 | 0.31221 |
|  | *Dcc* | -1.18188 | 0.62540 | 0.55648 |
|  | *Map2* | -0.97151 | -0.03891 | 1.01042 |
|  | *Nefl* | -1.09728 | 0.55118 | 0.54610 |
|  | *Ncan* | -0.62794 | -0.41916 | 1.04709 |
|  | *Mapt* | -0.75222 | -0.45337 | 1.20558 |
|  | *Robo2* | -1.05882 | -0.01654 | 1.07536 |
|  | *Nrcam* | -0.92945 | -0.10646 | 1.03591 |
|  | *Slit1* | -0.89798 | -0.08216 | 0.98015 |
|  | *Cntn2* | -1.03875 | 1.13088 | -0.09213 |
|  | *NeuN* | -0.81708 | -0.29379 | 1.11087 |
| **Neural functionality** | *Gad1* | -1.00821 | -0.12622 | 1.13444 |
|  | *Nrxn1* | -0.67725 | -0.50283 | 1.18008 |
|  | *Gria1* | -0.07504 | -0.86474 | 0.93978 |
|  | *Epb41l1* | -1.21738 | 0.28530 | 0.93209 |
|  | *Lin7b* | -1.18909 | 0.28081 | 0.90828 |
|  | *Stx1a* | -0.84712 | -0.30439 | 1.15151 |
|  | *Stxbp1* | -0.94119 | 0.04573 | 0.89546 |
|  | *Gabrg2* | -0.71155 | -0.42345 | 1.13499 |
|  | *Gabrb2* | -0.77367 | -0.37076 | 1.14443 |
|  | *Gabrb1* | -0.67877 | -0.61733 | 1.29609 |
|  | *Gria3* | -0.78765 | -0.21978 | 1.00743 |
|  | *Grin3a* | -1.00240 | -0.19818 | 1.20058 |
|  | *Polr2d* | -0.69669 | 0.16305 | 0.53365 |
|  | *Pnoc* | -0.71284 | -0.33449 | 1.04733 |
|  | *Chrm2* | -0.79615 | -0.35936 | 1.15551 |
|  | *Sst* | -1.12414 | 0.54812 | 0.57602 |
|  | *Kcnf1* | -0.85376 | -0.33356 | 1.18732 |
|  | *Slc8a3* | -0.82269 | -0.26213 | 1.08482 |
|  | *Camk2a* | -1.00240 | -0.19818 | 1.20058 |
|  | *Atp1a3* | -0.47890 | -0.60043 | 1.07932 |
| **Regulation of pluripotency** | *Cdh1* | 1.20724 | -0.82312 | -0.38412 |
|  | *Pou5f1* | 1.17325 | -0.55129 | -0.62196 |
|  | *Epha1* | 1.29516 | -0.68391 | -0.61125 |
|  | *Zscan10* | 1.23365 | -0.57073 | -0.66292 |
|  | *Dppa2* | 0.65267 | 0.52394 | -1.17661 |
|  | *Lefty1* | 0.30001 | 0.93764 | -1.23765 |
|  | *Foxh1* | 1.28393 | -0.53891 | -0.74502 |
|  | *Tdgf1* | 1.01904 | 0.18083 | -1.19988 |
| **Cell cycle progression** | *Blm* | 0.88982 | 0.05766 | -0.94748 |
|  | *Dna2* | 0.74575 | 0.45425 | -1.20000 |
|  | *Plk4* | 1.12573 | -0.02252 | -1.10321 |
|  | *Pola2* | 0.97832 | -0.18716 | -0.79115 |
|  | *Top2a* | 0.72343 | 0.59172 | -1.31515 |
|  | *Cdca8* | 0.43051 | 0.79261 | -1.22312 |
|  | *Fancd2* | 1.11820 | -0.32721 | -0.79099 |
|  | *Mcm2* | 1.07244 | 0.05520 | -1.12764 |
|  | *Mcm7* | 0.45730 | 0.63051 | -1.08781 |
|  | *Rfc3* | 0.85555 | 0.36081 | -1.21636 |
| **Notch, Wnt and Shh signaling** | *Hs3st3b1* | -1.19998 | 0.93009 | 0.26989 |
|  | *Hes5* | -0.98894 | 0.27639 | 0.71255 |
|  | *Hs3st3a1* | -1.16522 | 0.90905 | 0.25618 |
|  | *Shh* | -0.59074 | -0.57499 | 1.16573 |
|  | *Notch1* | -1.02893 | 0.71511 | 0.31382 |
|  | *Nog* | -0.68349 | -0.42008 | 1.10357 |
|  | *Emx2* | -0.81244 | -0.34007 | 1.15251 |
|  | *Ascl1* | -1.18589 | 0.81725 | 0.36865 |

**2.2.3 Supplementary Table 3: Bulk RNA transcriptomics**

<https://open.scayle.es/dataset/alonso-olivares-et-al-2024>
